# Supplementary material for: Single Cell Transcriptomic and Chromatin Profiles Suggest Layer Vb Is the Only Layer With Shared Excitatory Cell Types in the Medial and Lateral Entorhinal Cortex
Source: Front Neural Circuits. 2022 Jan 26;15:806154. doi: 10.3389/fncir.2021.806154 (PMC8826650; doi:10.3389/fncir.2021.806154)
Supplement: Supplementary file 14 [file Data_Sheet_1.ZIP › LEC_Sup_vs_MEC_Sup_Motifs/homerResults/motif3.info.html]

Motif 3

## Information for 5-ATTAATMAGC (Motif 3)

G
T
C
A
G
C
A
T
A
C
G
T
C
G
T
A
T
C
G
A
C
G
A
T
G
T
A
C
C
G
T
A
C
A
T
G
G
T
A
C
  
Reverse Opposite:
